# Camrelizumab plus famitinib for advanced or metastatic urothelial carcinoma after platinum-based therapy: data from a multicohort phase 2 study

**DOI:** 10.1136/jitc-2021-004427

**Published:** 2022-05-10

**Authors:** Yuan-Yuan Qu, Zhongquan Sun, Weiqing Han, Qing Zou, Nianzeng Xing, Hong Luo, Xuepei Zhang, Chaohong He, Xiao-Jie Bian, Jinling Cai, Chunxia Chen, Quanren Wang, Ding-Wei Ye

**Affiliations:** 1 Department of Urology, Fudan University Shanghai Cancer Center, Shanghai, China; 2 Department of Oncology, Shanghai Medical College, Fudan University, Shanghai, China; 3 Department of Urology Surgery, Huadong Hospital Affiliated to Fudan University, Shanghai, China; 4 Department of Urology Surgery, Hunan Cancer Hospital, Changsha, China; 5 Department of Urology Surgery, Jiangsu Cancer Hospital, Nanjing, China; 6 Department of Urology Surgery, Cancer Hospital Chinese Academy of Medical Sciences, Beijing, China; 7 Department of Urology Surgery, Chongqing Cancer Hospital, Chongqing, China; 8 Department of Urology Surgery, The First Affiliated Hospital of Zhengzhou University, Zhengzhou, China; 9 Department of Urology Surgery, Henan Cancer Hospital, Zhengzhou, China; 10 Clinical Research and Development, Jiangsu Hengrui Pharmaceuticals Co., Ltd, Shanghai, China

**Keywords:** Urologic neoplasms, Urinary Bladder Neoplasms, Therapies, Investigational, Programmed Cell Death 1 Receptor, Immunotherapy

## Abstract

**Background:**

Dual blockade of immune checkpoint and angiogenesis is an effective strategy for multiple cancers. Camrelizumab is a monoclonal antibody against PD-1, and famitinib is a multitargeted receptor tyrosine kinase inhibitor with antiangiogenesis and antiproliferation activities against tumor cells. We conducted an open-label, multicenter phase 2 basket study of camrelizumab and famitinib in eight cohorts of genitourinary or gynecological cancers. Here, findings in cohort of advanced or metastatic urothelial carcinoma with platinum-progressive disease (cohort 2) are presented.

**Methods:**

Patients who had progressed after platinum-based chemotherapy for advanced or metastatic disease or had progressed within 12 months after completion of platinum-based (neo)adjuvant therapy were given camrelizumab (200 mg intravenously every 3 weeks) plus famitinib (20 mg orally once daily). Primary endpoint was objective response rate (ORR) per Response Evaluation Criteria in Solid Tumors version 1.1.

**Results:**

Totally, 36 patients were recruited. With a median duration from enrollment to data cut-off of 11.9 months (range 6.1–28.5), ORR was 30.6% (95% CI 16.3% to 48.1%). Median duration of response (DoR) was 6.3 months (95% CI 2.1 to not reached). Median progression-free survival (PFS) was 4.1 months (95% CI 2.2 to 8.2), and median overall survival (OS) was 12.9 months (95% CI 8.8 to not reached). Patients with bladder cancer (n=18) had numerically better outcomes, with an ORR of 38.9% (95% CI 17.3% to 64.3%) and a median PFS of 8.3 months (95% CI 4.1 to not reached). Median DoR and OS in this subpopulation had not been reached with lower limit of 95% CI of 4.2 months for DoR and 11.3 months for OS, respectively. Of 36 patients, 22 (61.1%) had grade 3 or 4 treatment-related adverse events, mainly decreased platelet count and hypertension.

**Conclusions:**

Camrelizumab plus famitinib showed potent antitumor activity in advanced or metastatic urothelial carcinoma patients after platinum-based chemotherapy. Patients with bladder cancer seemed to have better response to this combination.

**Trial registration number:**

NCT03827837.

**Key messages**

**What is already known on this topic**

- Immune checkpoint inhibitors (ICIs) were approved as monotherapy for locally advanced or metastatic urothelial carcinoma after platinum-based chemotherapy.

**What this study adds**

- We assessed addition of famitinib (a multitargeted receptor tyrosine kinase inhibitor exhibiting antiangiogenesis and antiproliferation activities) to camrelizumab (an ICI against PD-1) in advanced or metastatic urothelial carcinoma patients with platinum-progressive disease. The combination demonstrated promising clinical efficacy with no unexpected toxicities, especially in patients with bladder cancer.

**How this study might affect research, practice or policy**

- Camrelizumab plus famitinib might provide another choice for platinum-progressive, advanced or metastatic urothelial carcinoma. This study provides rationale for further study of this combination in large-scare phase 3 study.

## Background

Urothelial carcinoma is the most common urinary cancer worldwide that threatens the survival and quality of life. Despite local therapy, approximately one-third of patients will relapse and develop metastatic diseases.^1 2^ Additionally, about 5% of patients have distant metastases at initial diagnosis.^3^

Prognosis for patients with locally advanced or metastatic urothelial carcinoma is dismal. Platinum-based therapy is the current first-line standard of care. Around half of patients responded to cisplatin-containing or carboplatin-containing regimens, and the median overall survival (OS) was less than 16 months.^4–8^ During the preimmunotherapy era, second-line salvage chemotherapy only achieved tumor response in 8.6%–13.9% of patients and showed a median OS of approximately 7 months.^9–12^ Since 2016, emergency of immune checkpoint inhibitors (ICIs) such as pembrolizumab, nivolumab, and avelumab monotherapy has revolutionized urothelial carcinoma care after failure of platinum-based chemotherapy, with 17%–21.1% of patients achieving an objective response and a median survival of 6.5–10.3 months.^11 13 14^ Nowadays, numerous combinatorial studies of an ICI with anti-angiogenic agent or chemotherapy or two ICIs are conducting to further improve the outcomes.

Camrelizumab is a humanized monoclonal antibody against PD-1, which selectively blocks the PD-L1–PD-1 axis and eventually inhibits the immune escape of tumor cells.^15^ Famitinib is a multitargeted receptor tyrosine kinase inhibitor (TKI) that exhibits potent activities toward stem-cell factor receptor (c-kit), VEGFR-2, and platelet-derived growth factor receptor β (PDGFRβ) with an IC50 value of 2.3, 4.7, and 6.6 nM, respectively, and also shows high inhibitory activities against other kinases including FMS-like tyrosine kinase-1/3 receptor (FLT1/3), VEGFR3, proto-oncogene tyrosine-protein kinase receptor (RET), and TAM family of kinases (AXL and MER).^16^ In addition to tumor angiogenesis and proliferation, these targets are involved in immune suppression pathways^17–20^; thus famitinib has the potential to enhance the antitumor immune response to camrelizumab. In this context, we initiated a multicohort phase 2 study of camrelizumab and famitinib as monotherapy or combination therapy for genitourinary or gynecological cancers. Here, we present the results of camrelizumab plus famitinib in the cohort of checkpoint inhibitor-naïve, platinum-progressive patients with advanced or metastatic urothelial carcinoma (cohort 2).

## Methods

### Study design and patients

This open-label, multicenter, basket phase 2 study of camrelizumab and famitinib was composed of eight cohorts in genitourinary or gynecological cancers. The overall study design had been published, and results for camrelizumab plus famitinib in the cohort of advanced or metastatic renal cell carcinoma (cohort 1) and cohort of platinum-resistant recurrent ovarian cancer (cohort 3) had been reported separately.^21 22^ In the cohort 2, eligible patients were aged 18–75 years, had pathological or cytological evidence of metastatic or surgically unresectable locally advanced urothelial carcinoma, had progression after platinum-based chemotherapy for advanced or metastatic disease or had progression within 12 months after completion of platinum-based adjuvant or neoadjuvant therapy, and had received one or two lines of systemic therapy for advanced or metastatic disease. Mixed histology that showed predominantly transitional-cell features was also eligible. Besides, patients should have an Eastern Cooperative Group performance status of 0 or 1, at least one measurable disease based on Response Evaluation Criteria in Solid Tumors (RECIST) version 1.1, a life expectancy of 12 weeks or more, and adequate hematological, hepatic, and renal function. Key exclusion criteria included known active or a history of autoimmune disease; use of immunosuppressant or systemic hormone with 2 weeks before study; poorly controlled hypertension; untreated central nervous system metastases; radiological evidence of tumor invading major blood vessels; abnormal coagulation function, bleeding susceptibility or receiving thrombolysis or anticoagulation therapy. Prior chemotherapy within 4 weeks before study was not permitted. Prior surgery or palliative radiotherapy must be completed at least 2 weeks before study. Prior anti-PD-1/anti-PD-L1/anti-CTLA-4 antibodies was not allowed.

### Procedures

Our previous data showed that camrelizumab 200 mg every 3 weeks by intravenous infusion combined with oral famitinib 20 mg once daily was well tolerated.^23^ Hence, all patients in this cohort received camrelizumab 200 mg every 3 weeks plus famitinib 20 mg once daily until confirmed disease progression (except quick radiological progression and clinical progression), unacceptable toxicity, patient decision or withdrawal of consent, withdrawal by the investigator, or lost to follow-up, whichever occurred first. Patients with RECIST-defined progression and a clinically stable status could continue study therapy at the discretion of the investigator. The maximum total camrelizumab exposure was 2 years. Interruptions of camrelizumab or famitinib and dose reductions of famitinib were permitted to manage toxic events.

### Endpoints and assessments

The primary endpoint was objective response rate (ORR), defined as the percentage of patients with a best overall response of confirmed complete response (CR) or partial response (PR) according to RECIST version 1.1. Secondary endpoints were disease control rate (DCR), time to response (TTR), duration of response (DoR), progression-free survival (PFS), OS, 12-month OS rate, and safety.

Tumor responses were assessed by the investigator, at baseline and then every three cycles, according to RECIST version 1.1. CRs or PRs were confirmed with a repeat scan at least 4 weeks after the initial response. After treatment discontinuation, patients were followed-up for survival status every 2 months. Vital sign, laboratory tests, 12-lead electrocardiograms, echocardiography, and adverse events (AEs) were monitored for safety assessments. AEs were assessed and graded according to the National Cancer Institute Common Terminology Criteria for Adverse Events (V.4.0) until 30 days after the last dose. Serious AEs (SAEs) and treatment-related AEs (TRAEs) were collected until 90 days after the last dose.

The PD-L1 was centrally tested using archival or fresh tumor tissues by PD-L1 IHC 22C3 pharmDx test (Dako, Carpinteria, California, USA). PD-L1 expression was calculated as Combined Positive Score (CPS), defined as the number of PD-L1 staining cells (tumor cells, lymphocytes, and macrophages) out of the total number of tumor cells, multiplied by 100.

### Statistical analyses

For this cohort, sample size was calculated using an adaptive two-stage design.^24^ An ORR of 15% was considered ineffective, 25% was considered low desirable response, and 35% was considered high desirable response. Assuming ORR as specified, a power of 80% for a high desirable response and 70% for a low desirable response, and a two-sided α level of 0.1, 22 patients would be enrolled at stage one; at stage two, enrollment would be terminated or extended to 53 or 33 depending on the observed response rate at stage one. Study treatment was considered effective if at least 12 of the 53 patients or at least 8 of the 33 patients responded.

At stage 1, 7 of the 22 patients in this cohort achieved objective responses, and thus enrollment of stage two was initiated. Efficacy was assessed in all patients with at least one dose of the study treatment. Safety was assessed in all patients who received at least one dose of study treatment and had at least one post-baseline assessments. ORR and DCR were calculated with their 95% CIs being estimated by the Clopper-Pearson method. Time-to-event endpoints including TTR, DoR, PFS, and OS were estimated with the Kaplan-Meier method, with the 95% CIs for median being calculated with the Brookmeyer and Crowley method and the 95% CIs for survival rates being calculated by means of log-log transformation (on the basis of normal approximation) with back transformation to CIs on the untransformed scale.

## Results

### Patient disposition and baseline characteristics

From January 23, 2019 to December 14, 2020, a total of 36 patients with urothelial carcinoma from eight study sites in China were enrolled and treated with camrelizumab combined with famitinib. Median patient age was 62.5 years (range 43.0–79.0), and 28 (77.8%) patients were males (table 1). Primary tumors were commonly found in the urinary bladder (n=18, 50.0%), followed by the renal pelvic (n=10, 27.8%). Twenty (55.6%) patients had at least two sites of metastases; and the most common metastasis site was lung (n=17, 47.2%). All patients had received platinum-based therapy.

**Table 1:** Patient demographics and baseline characteristics

	Patients (N=36)
Median age (range), years	62.5 (43.0–79.0)
Sex	
Male	28 (77.8%)
Female	8 (22.2%)
ECOG performance status	
0	11 (30.6%)
1	25 (69.4%)
No. of organs of metastases	
1	14 (38.9%)
2	6 (16.7%)
>2	14 (38.9%)
Common visceral diseases	
Lung metastases	17 (47.2%)
Bone metastases	8 (22.2%)
Liver metastases	8 (22.2%)
Subsite of primary tumor	
Bladder	18 (50.0%)
Renal pelvis	10 (27.8%)
Ureter	5 (13.9%)
Mixed	2 (5.6%)
Urethra	1 (2.8%)
Prior surgery for primary tumor	29 (80.6%)
Prior platinum-based therapies	36 (100%)
Adjuvant therapy	1 (2.8%)
One line	31 (86.1%)
Two lines	4 (11.1%)
Previous systemic therapy	
Platinum in neoadjuvant or adjuvant settings*	2 (5.6%)
Platinum in advanced or metastatic settings	35 (97.2%)
Cisplatin-based regimen only	24 (66.7%)
Carboplatin-based regimen only	4 (11.1%)
Both cisplatin-based and carboplatin-based regimens	1 (2.8%)
Other platinum-based regimen	6 (16.7%)
PD-L1 CPS in 27 evaluable patients	
<1	19 (70.4%)
≥1	8 (29.6%)

Data are n (%) unless stated otherwise.

*One patient had received platinum-based regimen both in neoadjuvant or adjuvant setting and advanced or metastatic setting.

CPS, Combined Positive Score; ECOG, Eastern Cooperative Group.

As of June 8, 2021, the median follow-up duration from enrollment to data cut-off was 11.9 months (range, 6.1–28.5). The median cycle of camrelizumab received by patients was 10 (range 2–36), and the median exposure of famitinib was 28.4 weeks (range, 1.9–111.6). At the time of data cut-off, 11 (30.6%) patients were still receiving study treatment. The most common reason for discontinuation was disease progression (n=19, 52.8%), followed by withdrawal by patients, investigator decision, and AEs (n=2, 5.6% for each).

### Efficacy in all patients

**Figure 1:**
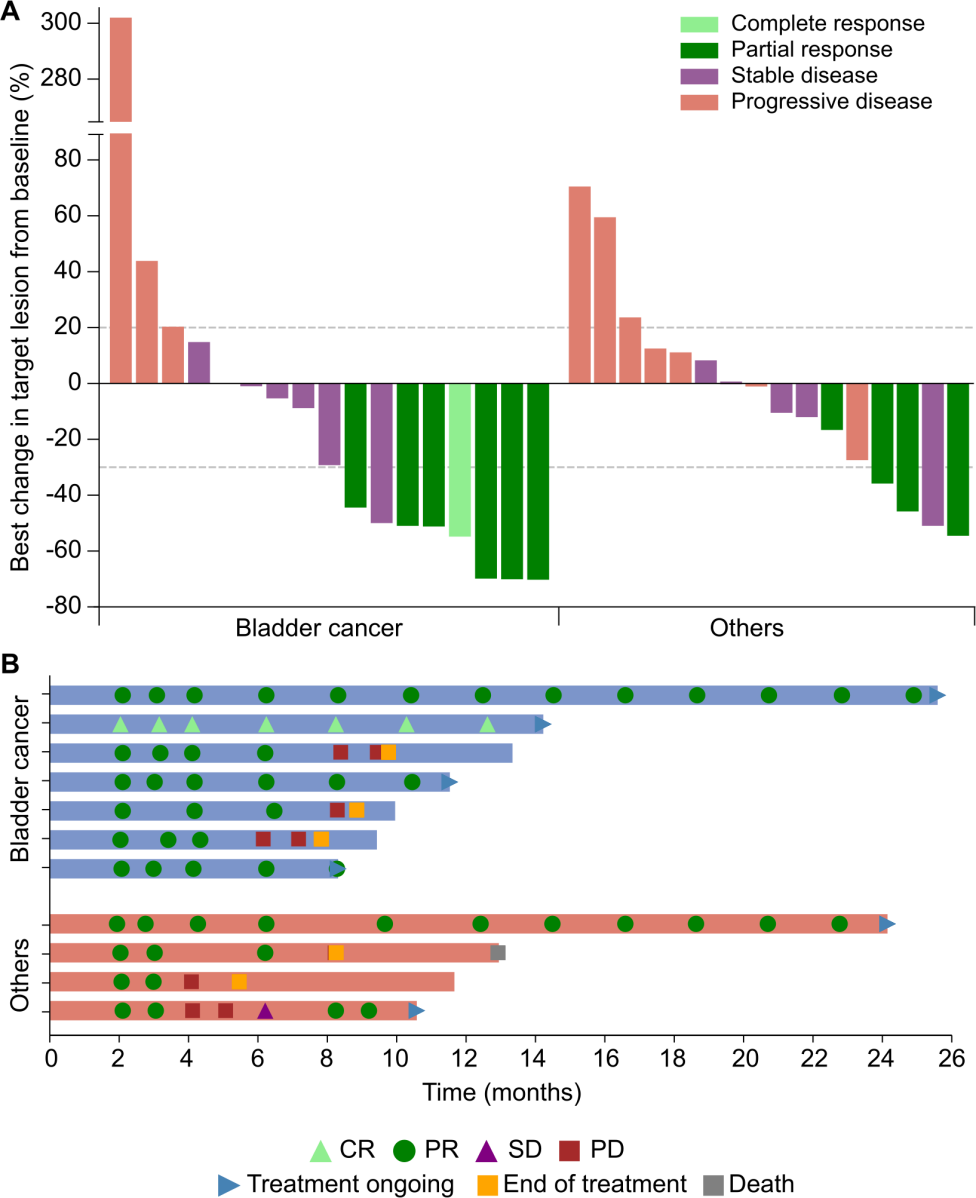
Clinical activity. (A) Best change in target lesion from baseline in all evaluable patients; (B) tumor responses over time. For the patient who achieved with complete response (CR), the target lesions included pathological lymph nodes, and thus the change in target lesions was not −100%. PR, partial response; SD, stable disease; PD, progressive disease.

As shown in figure 1A, 21 of the 33 (63.6%) patients who had postbaseline target lesion assessment showed tumor shrinkage. Tumor responses in all 36 patients were summarized in table 2. Objective response was achieved in 30.6% (95% CI 16.3% to 48.1%) of patients, including one (2.8%) CR and 10 (27.8%) PRs. Stable disease was observed in 12 (33.3%) patients, and DCR was 63.9% (95% CI 46.2% to 79.2%).

**Table 2:** Tumor responses

	All patients (N=36)	Primary tumor type	
		Bladder cancer (n=18)	Other types (n=18)
Best overall response, n (%)			
Complete response	1 (2.8)	1 (5.6)	0
Partial response	10 (27.8)	6 (33.3)	4 (22.2)
Stable disease	12 (33.3)	7 (38.9)	5 (27.8)
Progressive disease	10 (27.8)	3 (16.7)	7 (38.9)
Not evaluable	3 (8.3)	1 (5.6)	2 (11.1)
Objective response rate, % (95% CI)	30.6 (16.3 to 48.1)	38.9 (17.3 to 64.3)	22.2 (6.4 to 47.6)
Disease control rate, % (95% CI)	63.9 (46.2 to 79.2)	77.8 (52.4 to 93.6)	50.0 (26.0 to 74.0)
Median time to response (range), months	2.1 (1.9–2.1)	2.1 (2.0–2.1)	2.1 (1.9–2.1)
Duration of response			
Patients with ongoing response, n/N (%)	5/11 (45.5)	4/7 (57.1)	1/4 (25.0)
Median (95% CI)	6.3 (2.1 to NR)	NR (4.2 to NR)	4.2 (2.1 to NR)
6-month rate	72.7% (37.1 to 90.3)	85.7% (33.4 to 97.9)	50.0% (5.8 to 84.5)

NR, not reached.

Of the 11 responders, the responses were ongoing in 5 (45.5%) patients (figure 1B). The median DoR was 6.3 months (95% CI 2.1 to not reached), and DoR rate at 6 months was 72.7% (95% CI 37.1% to 90.3%). The median TTR was 2.1 months (range 1.9–2.1).

**Figure 2:**
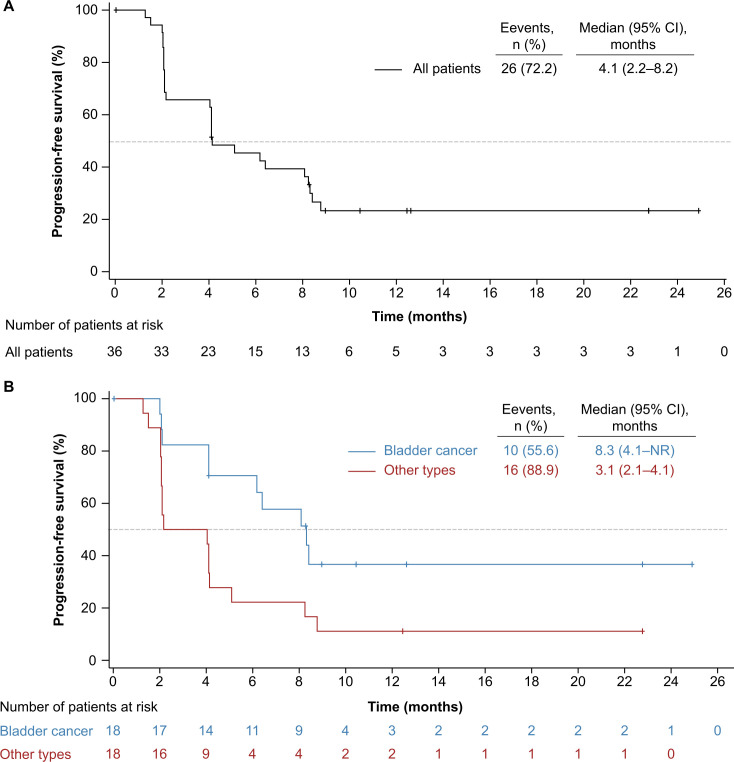
Kaplan-Meier estimates of progression-free survival. (A) All patients; (B) subgroup by primary tumor types. NR, not reached.

**Figure 3:**
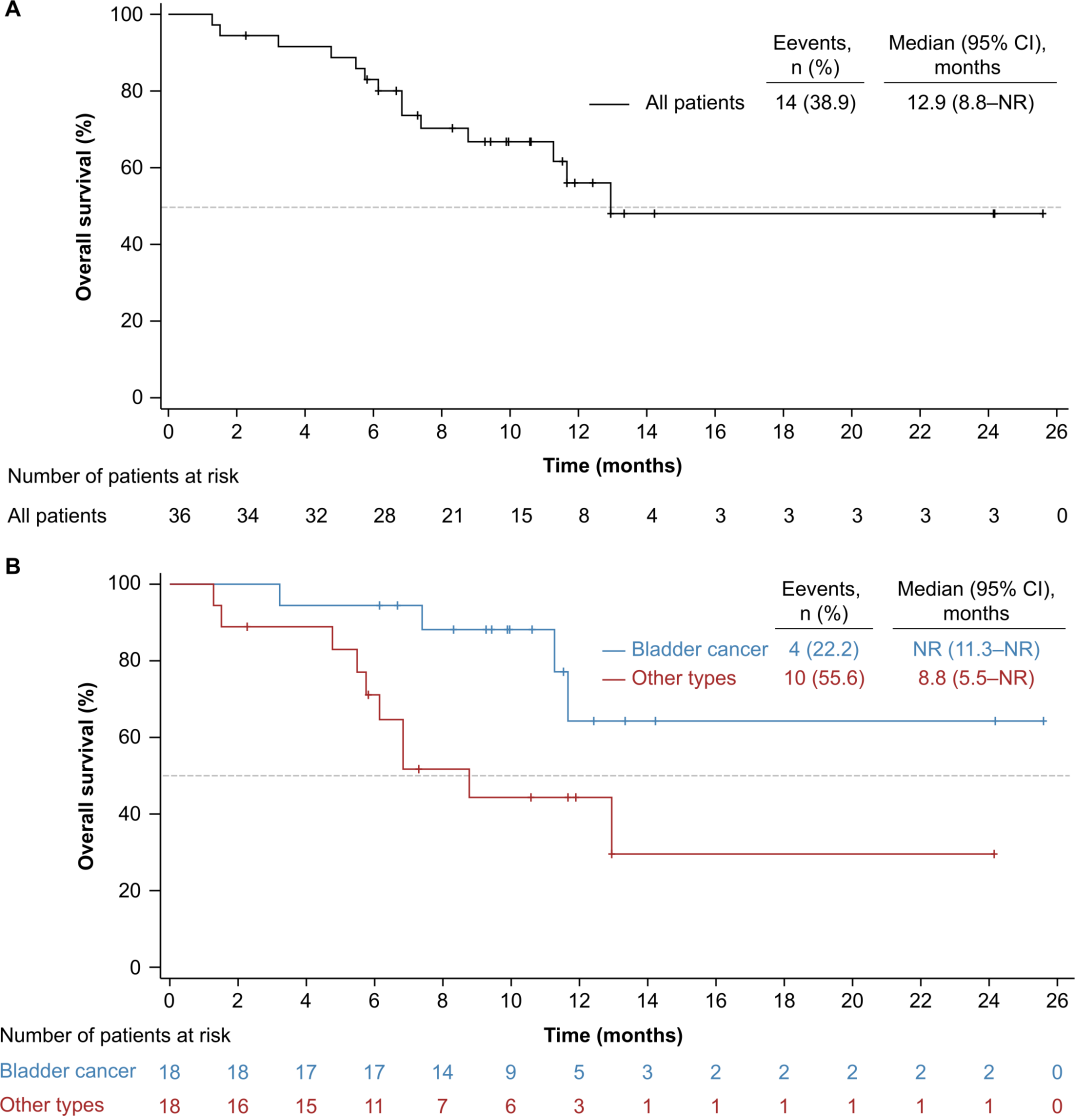
Kaplan-Meier estimates of overall survival. (A) All patients; (B) subgroup by primary tumor types. NR, not reached.

As of cut-off date, 26 (72.2%) events of disease progression or deaths occurred. The median PFS was 4.1 months (95% CI 2.2 to 8.2; figure 2A), and PFS rate at 6 months was 45.4% (95% CI 28.5% to 60.8%). There were 14 (38.9%) deaths occurred. The median OS was 12.9 months (95% CI 8.8 to not reached; figure 3A), and 12-month OS rate was 56.0% (95% CI 35.0% to 72.6%).

### Efficacy in subgroup by the primary tumor type

ORR was 38.9% (95% CI 17.3% to 64.3%) in the 18 patients with bladder cancer vs 22.2% (95% CI 6.4% to 47.6%) in the 18 patients with other urothelial cancer, and DCR was 77.8% (95% CI 52.4% to 93.6%) vs 50.0% (95% CI 26.0% to 74.0%), respectively (table 2).

At the time of data cut-off, four of seven (57.1%) responders in the bladder cancer subgroup and one of four (25.0%) responders in the other urothelial cancer subgroup continued to have a response. The median DoR in patients with bladder cancer had not been reached yet (95% CI 4.2 to not reached), while in patients with other urothelial cancers, the median DoR was 4.2 months (95% CI 2.1 to not reached). Six-month DoR rate was 85.7% (95% CI 33.4% to 97.9%) versus 50.0% (95% CI 5.8% to 84.5%). The median TTR was similar between the bladder cancer and other urothelial cancer subgroups (table 2).

A total of 10 (55.6%) and 16 (88.9%) events of disease progression or death occurred in the bladder cancer and other urothelial cancer subgroups. The median PFS was 8.3 months (95% CI 4.1 to not reached) vs 3.1 months (95% CI 2.1 to 4.1; figure 2B), and 6 months PFS rate was 70.6% (95% CI 43.1% to 86.6%) vs 22.2% (95% CI 6.9% to 42.9%), respectively. Four (22.2%) patients with bladder cancer and 10 (55.6%) with other urothelial cancer died at cut-off date. The median OS had not been reached yet (95% CI 11.3 to not reached) in the bladder cancer subgroup and was 8.8 months (95% CI 5.5 to not reached) in the other urothelial cancer subgroup (figure 3B), and OS rate at 12 months was 64.3% (95% CI 27.2% to 86.1%) and 44.3% (95% CI 19.8% to 66.4%), respectively.

### Efficacy in subgroup by PD-L1 expression

Tumor biospecimens of 27 patients were available for PD-L1 expression assessment. Eight patients had PD-L1 CPS ≥1. ORR was 37.5% (95% CI 8.5% to 75.5%) in patients with PD-L1 CPS ≥1% and 31.6% (95% CI 12.6% to 56.6%) in patients with PD-L1 CPS<1.

### Safety

TRAEs of any grade occurred in all 36 patients (table 3), with the most common ones being proteinuria (n=25, 69.4%), decreased platelet count (n=24, 66.7%), anemia (n=20, 55.6%), palmar-plantar erythrodysesthesia (PPE) syndrome (n=15, 41.7%), and decreased white blood cell count (n=15, 41.7%). A total of 17 (47.2%) patients experienced grade 3 TRAEs; those occurring in more than 10% of patients were decreased platelet count and hypertension (n=5 each, 13.9%). Five (13.9%) patients had grade 4 TRAEs (decreased platelet count, n=2, 5.6%; hyperuricemia, n=2, 5.6%; increased blood creatinine, n=1, 2.8%).

**Table 3:** Summary of TRAEs

	All patients (N=36)
TRAEs	
Any grade	36 (100.0%)
Grade 3	17 (47.2%)
Grade 4	5 (13.9%)
Serious	7 (19.4%)
TRAEs leading to	
Camrelizumab interruption	14 (38.9%)
Camrelizumab discontinuation	1 (2.8%)
Famitinib dose reduction/interruption	21 (58.3%)
Famitinib interruption	20 (55.6%)
Famitinib dose reduction	9 (25.0%)
Famitinib discontinuation	1 (2.8%)
Deaths	1 (2.8%)

Common TRAEs*	Any grade	Grade 3	Grade 4
Proteinuria	25 (69.4%)	2 (5.6%)	0
Platelet count decreased	24 (66.7%)	5 (13.9%)	2 (5.6%)
Anemia	20 (55.6%)	3 (8.3%)	0
PPE syndrome	15 (41.7%)	3 (8.3%)	0
WBC count decreased	15 (41.7%)	1 (2.8%)	0
Hypertension	13 (36.1%)	5 (13.9%)	0
Blood creatinine increased	11 (30.6%)	0	1 (2.8%)
Neutrophil count decreased	11 (30.6%)	1 (2.8%)	0
Diarrhea	11 (30.6%)	1 (2.8%)	0
ALT increased	10 (27.8%)	1 (2.8%)	0
Asthenia	10 (27.8%)	0	0
AST increased	9 (25.0%)	0	0
Decreased appetite	9 (25.0%)	0	0
Hyperuricemia	7 (19.4%)	1 (2.8%)	2 (5.6%)
GGT increased	7 (19.4%)	2 (5.6%)	0
Hypertriglyceridemia	6 (16.7%)	1 (2.8%)	0
RCEP	6 (16.7%)	1 (2.8%)	0
Blood pressure increased	6 (16.7%)	0	0
Hypokalemia	4 (11.1%)	2 (5.6%)	0

Data are shown in n (%).

*TRAEs of all grades occurring in at least 15% of patients, grade 3 TRAEs occurring in at least 5% of patients, and all grade 4 TRAEs are listed. One patient died due to TRAE, multiple organ dysfunction syndrome.

ALT, alanine aminotransferase; AST, aspartate aminotransferase; GGT, gamma-glutamyltransferase; PPE, palmar-plantar erythrodysesthesia; RCEP, reactive capillary endothelial proliferation; TRAE, treatment-related adverse event; WBC, white blood cell.

Treatment-related SAEs occurred in seven (19.4%) patients (online supplemental table S1), including decreased platelet count (n=3, 8.3%) and multiple organ dysfunction syndrome, pyrexia, immune-mediated hepatitis, reactive capillary endothelial proliferation (RCEP), and myelosuppression (n=1 each, 2.8%). Totally, there were seven deaths due to AEs, among them only one was deemed to be treatment-related by the investigator. The patient died of multiple organ dysfunction syndrome.

10.1136/jitc-2021-004427.supp1 Supplementary data

One (2.8%) patient discontinued treatment because of TRAE (multiple organ dysfunction syndrome). TRAEs led to dose interruption of camrelizumab in 14 (38.9%) patients, with decreased platelet count, increased blood creatinine, thyroiditis, anemia, RCEP, and hypothyroidism occurring in more than one patient (online supplemental table S2). Twenty (55.6%) patients experienced at least one TRAE leading to famitinib interruption, mainly including decreased platelet count, anemia, hypertension, proteinuria, PPE syndrome, increased blood creatinine, thyroiditis, decreased white blood cell count, hypothyroidism, and diarrhea occurring in more than one patient (online supplemental table S2). TRAEs led to dose reduction of famitinib in nine (25.0%) patients, with PPE syndrome occurring in more than one patient.

Immune-related AEs (irAEs), regardless of whether they were attributed to study treatment by investigators, occurred in 6 (16.7%) of 36 patients, including hypothyroidism (n=2, 5.6%) and hyperthyroidism, autoimmune thyroiditis, immune-mediated hepatitis, immune-mediated hepatic disorder, pyrexia, asthenia, generalized edema, increased blood thyroid stimulating hormone, hypersensitivity, immune-mediated dermatitis, pruritus, and cheilitis (n=1 each, 2.8%).

RCEP was reported in 16.7% of patients (n=6). Majority of the events were grade 1 or 2 in severity (n=5, 13.9%), and only one (2.8%) patient had grade 3 RCEP.

## Discussion

In this phase 2 study, camrelizumab combined with famitinib was associated with promising antitumor activity in patients with advanced or metastatic urothelial carcinoma after platinum-based therapy, with an ORR of 30.6% (95% CI 16.3% to 48.1%), a median PFS of 4.1 months (95% CI 2.2 to 8.2), and a median OS of 12.9 months (95% CI 8.8 to not reached).

Urothelial carcinomas are considered immunogenic with high PD-L1 expression level and high somatic mutation burden,^25–27^ providing a theoretical basis for immunotherapy. In the clinical studies of ICI monotherapy after platinum-based therapy, pembrolizumab achieved tumor response in 21.1% (95% CI 16.4% to 26.5%) of patients, nivolumab in 19.6% (95% CI 15.0% to 24.9%), and avelumab in 17% (95% CI 11% to 24%).^11 13 14^ The proportion of patients respond to camrelizumab plus famitinib compared favorably with those reported data (approximately 10% higher). Of note, urothelial bladder cancer accounted for over 70% of patients enrolled in these studies of ICI monotherapy. Response to pembrolizumab or avelumab in this subpopulation with bladder cancer was not available; the ORR with nivolumab was 22% (95% CI 16% to 28%).^13^ In our study, 50.0% of the enrolled patients had urothelial carcinoma of bladder and exhibited an ORR of 38.9% (95% CI 17.3% to 64.3%). Our findings indicted antiangiogenic TKI famitinib as an attractive drug when combined with camrelizumab to augment immunotherapy response in urothelial carcinoma, especially in bladder cancer.

Shorter median DoR was indicated with PD-1 inhibitor combined with TKI compared with PD-1 inhibitor monotherapy (6.3 months with camrelizumab plus famitinib and 8.3 months with pembrolizumab plus ramucirumab), which might because some responders mainly benefited from the TKI considering that only 5.1 months or less was achieved by TKI alone.^28^ However, more patients responded to the combination, bringing prolonged survival benefit. In patients with advanced or metastatic urothelial carcinoma after platinum-based therapy, the median PFS was 2.1 months (95% CI 2.0 to 2.2) with pembrolizumab, 2.00 months (95% CI 1.87 to 2.63) with nivolumab, and 6.3 weeks (95% CI 6.0 to 10.1) with avelumab,^11 13 14^ with no improvement compared with historical data with single-drug chemotherapies in this setting.^9–12^ By combining with an antiangiogenic agent, camrelizumab plus famitinib attained a favorable median PFS of 4.1 months (95% CI 2.2 to 8.2). Also, clinical meaningful improvement in OS was observed (median, 12.9 months (95% CI 8.8 to not reached) with camrelizumab plus famitinib compared with 6.5–10.3 months with avelumab, nivolumab, or pembrolizumab). Notably, in the subpopulation with bladder cancer in our study, the median PFS was as high as 8.3 months (95% CI 4.1 to not reached), and the median OS had not been reached with lower limit of 95% CI of 11.3 months.

While upper-tract urothelial carcinoma (UTUC) share similar histological appearance with bladder urothelial carcinoma, they have differences in etiology, clinical phenotype, and molecular alterations. UTUC is a rare malignancy associated with an aggressive phenotype. About 60% of UTUC patients have muscle invasive disease at diagnosis and nearly 25% have regional metastasis.^29–31^ Asian patients seem to present with more advanced and higher-grade diseases compared with other ethnicities,^32^ which might enlarge the distinct outcomes of UTUC and bladder urothelial carcinoma in our study. Tumor genomic analysis showed higher *FGFR3* alterations in UTUC patients, and these patients could potentially benefit from FGFR3-targeted therapy.^33^ Patients with bladder urothelial carcinoma had higher PD-L1 than those with UTUC and thus were more likely to benefit from immunotherapy.^33^

Early-phase clinical trials have explored combinations in patients with advanced or metastatic urothelial carcinoma in the second-line setting. In a multicohort phase 1 a/b trial of ramucirumab plus pembrolizumab, despite manageable safety profile, no obviously favorable antitumor efficacy over ICI monotherapy was observed (ORR, 13% (95% CI 2.7 to 32.4); median PFS, 1.9 months (95% CI 1.2 to 2.8); median OS, 6.4 months (95% CI 2.5 to 18.7)).^34^ In two phase 2 studies involving combination of small-molecule TKI and ICI in cohort of in urothelial carcinoma, encouraging efficacy was reported. Lenvatinib plus pembrolizumab showed an ORR of 25% (95% CI 8.7% to 49.1%) and median PFS of 5.4 months (95% CI 1.3 to not reached),^35^ and cabozantinib plus durvalumab had an ORR of 37.5% (95% CI 15.2% to 64.6%).^36^ Our findings were comparable to these released data.

There are two phase 3 studies of the combinations as front-line treatment for urothelial carcinoma. The LEAP-011 study (NCT03898180) of first-line pembrolizumab plus lenvatinib was stopped early. In patients who were cisplatin-ineligible with PD-L1-positive tumors (CPS ≥10) or were ineligible to receive any platinum-based chemotherapy, no significant differences in median PFS and OS were found between pembrolizumab plus lenvatinib and pembrolizumab plus placebo.^37^ The MAIN-CAV study (NCT05092958) of maintenance cabozantinib plus avelumab after first-line platinum-based chemotherapy is ongoing and the results are expected.

Data from clinical trials of ICI monotherapy showed inconsistent results in terms of associations between response and PD-L1 expression in urothelial carcinoma,^11–14^ which may be caused by differences in procedure of tissue collection and fixation, antibody and assay used for PD-L1 test, definition of PD-L1 expression, and cut-off for PD-L1 positivity. For combination therapy, there was also no evidence supporting the use of PD-L1 as a biomarker in urothelial carcinoma.^34–36^ In this study, antitumor responses were seen irrespective of PD-L1 expression, even patients with PD-L1 CPS<1 achieved an ORR of 31.6% (95% CI 12.6% to 56.6%).

In line with our findings in the cohorts of advanced or metastatic renal cell carcinoma and ovarian cancer,^21 22^ no new safety concerns with camrelizumab plus famitinib were found in patients with advanced or metastatic urothelial carcinoma. Generally, with median duration from enrollment of 11.9 months, camrelizumab 200 mg every 3 weeks plus famitinib 20 mg once daily were tolerable and the AEs were manageable.

Most AEs that mainly attributed to famitinib such as proteinuria, hypertension, PPE syndrome, and hematological toxicities^38–40^ could be controlled by treatment interruption. Only 25% of patients had unsolved AEs that needed to reduce the dose of famitinib.

RCEP is the most common AE attributable to camrelizumab monotherapy occurring in 67%–97.3% of the patients in previous studies, but majority of them were grade 1 (nodules with a maximum diameter of ≤10 mm) or grade 2 (nodules with a maximum diameter of >10 mm) in severity.^15 41–46^ The events mainly occurred on skin of the head, face, and trunk, and most lesions were scattered on the skin, which was different from other common skin irAE such as rash. According to the morphology, RCEP could be divided into five types including ‘red-nevuslike’ ‘pearl-like’ ‘mulberry-like’ ‘patch-like’ and ‘tumor-like’, with the first two being the most common types.^47^ Growth of RCEP experienced a tripartite cycle of proliferation, plateau, and involution, and most lesions spontaneously resolved after discontinuation of camrelizumab. When combined with famitinib, only 16.7% of urothelial carcinoma patients in this study experienced RCEP. This was consistent with other studies involving camrelizumab in combination with a VEGFR inhibitor.^21 22 48 49^ It has been speculated that camrelizumab-induced reactivation of the immune response disrupts the balance between pro-angiogenic and anti-angiogenic growth factors, finally promoting vascular proliferation by releasing VEGF-A.^47^ Thus, combination with famitinib might inhibit the development of RCEP by blocking VEGF signal transduction.

Only one (2.8%) patient discontinued treatment owing to TRAE. The patient was male aged 64 years and had high-grade urothelial carcinoma of the ureter. He received two cycles of camrelizumab plus famitinib and died for multiple organ dysfunction syndrome that was judged possibly related to study treatment by investigator. But the possible reason for death was noted to be progressive disease. As there are no reported evidence of causality between multiple organ dysfunction syndrome and camrelizumab or famitinib, and no deaths due to multiple organ dysfunction syndrome occurred in other cohorts of this study, further assessment is required.

Due to the exploratory nature of this phase 2 study, the major limitation of this study is lack of a control arm. It is hard to contextualize our findings relative to approved ICI monotherapy. Besides, due to small sample size, bladder cancer and PD-L1 subgroup findings need further investigation. Aside from PD-L1 expression, translational and biomarker analyses were not done as it was not mandatory for patients to provide the tumor sample. A company-sponsored, randomized, controlled phase 3 clinical trial is planning to assess this combination in patients with untreated advanced or metastatic urothelial carcinoma and to explore candidate prognostic biomarkers.

In conclusion, this study demonstrated the promising clinical activity and controllable safety of camrelizumab combined with famitinib in patients with advanced or metastatic urothelial carcinoma after platinum-based therapy. Patients with bladder cancer seemed to have better response to this combination. Our findings support further investigation of this combination in large-scare phase 3 study.

## Data Availability

Data are available on reasonable request. Data are available on reasonable request. The data that support the findings of this study are available from the corresponding author on reasonable request.
