# Supplementary material for: Camrelizumab plus famitinib for advanced or metastatic urothelial carcinoma after platinum-based therapy: data from a multicohort phase 2 study
Source: J Immunother Cancer. 2022 May 10;10(5):e004427. doi: 10.1136/jitc-2021-004427 (PMC9092172; doi:10.1136/jitc-2021-004427)
Supplement: Supplementary data [file jitc-2021-004427supp001.pdf]

Supplementary Table S1. Serious treatment-related adverse events

	**Any grade**	**Grade ≥3**
**Any**	7 (19.4%)	7 (19.4%)
Platelet count decreased	3 (8.3%)	3 (8.3%)
Multiple organ dysfunction syndrome*	1 (2.8%)	1 (2.8%)
Pyrexia	1 (2.8%)	1 (2.8%)
Immune-mediated hepatitis	1 (2.8%)	1 (2.8%)
Reactive capillary endothelial proliferation	1 (2.8%)	1 (2.8%)
Myelosuppression	1 (2.8%)	0

Data are shown in n (%).

*Totally, one death was deemed treatment related by the investigator. The patient was male aged 64 years and had high-grade urothelial carcinoma of the ureter. He received two cycles of camrelizumab plus famitinib and died for multiple organ dysfunction syndrome that was judged possibly related to study treatment by investigator. But the possible reason for death was noted to be progressive disease. As there are no reported evidence of causality between multiple organ dysfunction syndrome and camrelizumab or famitinib, and no deaths due to multiple organ dysfunction syndrome occurred in other cohorts of this study, further assessment is required.

Qu Y-Y, *et al. J Immunother Cancer* 2022; 10:e004427. doi: 10.1136/jitc-2021-004427

Supplementary Table S2. TRAEs leading to dose modification or treatment discontinuation

	**Any grade**	**Grade ≥3**
**TRAEs leading to interruption of camrelizumab**	14 (38.9%)	10 (27.8%)
Platelet count decreased	5 (13.9%)	4 (11.1%)
Blood creatinine increased	3 (8.3%)	1 (2.8%)
Thyroiditis	2 (5.6%)	1 (2.8%)
Anemia	2 (5.6%)	1 (2.8%)
Reactive capillary endothelial proliferation	2 (5.6%)	1 (2.8%)
Hypothyroidism	2 (5.6%)	0
Immune-mediated hepatitis	1 (2.8%)	1 (2.8%)
Immune-mediated hepatic disorder	1 (2.8%)	1 (2.8%)
Pyrexia	1 (2.8%)	1 (2.8%)
Hyperkalemia	1 (2.8%)	1 (2.8%)
White blood cell count decreased	1 (2.8%)	0
Alanine aminotransferase increased	1 (2.8%)	0
Aspartate aminotransferase increased	1 (2.8%)	0
Blood urea increased	1 (2.8%)	0
Myelosuppression	1 (2.8%)	0
Face edema	1 (2.8%)	0
Hyperuricemia	1 (2.8%)	0
Immune-mediated dermatitis	1 (2.8%)	0
Pruritus	1 (2.8%)	0
Abdominal pain	1 (2.8%)	0
Diarrhea	1 (2.8%)	0
**TRAEs leading to discontinuation of camrelizumab**	1 (2.8%)	1 (2.8%)

Qu Y-Y, *et al. J Immunother Cancer* 2022; 10:e004427. doi: 10.1136/jitc-2021-004427

Multiple organ dysfunction syndrome	1 (2.8%)	1 (2.8%)
**TRAEs leading to interruption of famitinib**	20 (55.6%)	15 (41.7%)
Platelet count decreased	7 (19.4%)	5 (13.9%)
Anemia	5 (13.9%)	3 (8.3%)
Hypertension	3 (8.3%)	3 (8.3%)
Proteinuria	3 (8.3%)	1 (2.8%)
Palmar-plantar erythrodysesthesia syndrome	2 (5.6%)	2 (5.6%)
Blood creatinine increased	2 (5.6%)	1 (2.8%)
Thyroiditis	2 (5.6%)	1 (2.8%)
White blood cell count decreased	2 (5.6%)	0
Hypothyroidism	2 (5.6%)	0
Diarrhea	2 (5.6%)	0
Alanine aminotransferase increased	1 (2.8%)	1 (2.8%)
Stomatitis	1 (2.8%)	1 (2.8%)
Immune-mediated hepatitis	1 (2.8%)	1 (2.8%)
Immune-mediated hepatic disorder	1 (2.8%)	1 (2.8%)
Pyrexia	1 (2.8%)	1 (2.8%)
Hyperkalemia	1 (2.8%)	1 (2.8%)
Pneumonitis	1 (2.8%)	1 (2.8%)
Aspartate aminotransferase increased	1 (2.8%)	0
Blood urea increased	1 (2.8%)	0
Myelosuppression	1 (2.8%)	0
Abdominal pain	1 (2.8%)	0
Rectal hemorrhage	1 (2.8%)	0
Immune-mediated dermatitis	1 (2.8%)	0
Rash	1 (2.8%)	0
Pruritus	1 (2.8%)	0
Face edema	1 (2.8%)	0
Hyperuricemia	1 (2.8%)	0

**TRAEs leading to reduction of famitinib**	9 (25.0%)	4 (11.1%)
Palmar-plantar erythrodysesthesia syndrome	3 (8.3%)	1 (2.8%)
Diarrhea	1 (2.8%)	1 (2.8%)
Hypokalemia	1 (2.8%)	1 (2.8%)
Platelet count decreased	1 (2.8%)	1 (2.8%)
Proteinuria	1 (2.8%)	1 (2.8%)
Stomatitis	1 (2.8%)	0
Immune-mediated hepatic disorder	1 (2.8%)	0
Myelosuppression	1 (2.8%)	0
**TRAEs leading to discontinuation of famitinib**	1 (2.8%)	1 (2.8%)
Multiple organ dysfunction syndrome	1 (2.8%)	1 (2.8%)

Data are shown in n (%). TRAEs, treatment-related adverse events.

Qu Y-Y, *et al. J Immunother Cancer* 2022; 10:e004427. doi: 10.1136/jitc-2021-004427

Supplementary Table S3. Immune-related adverse events

	**Any grade**	**Grade 3**
**Any**	6 (16.7%)	3 (8.3%)
Hypothyroidism	2 (5.6%)	0
Hyperthyroidism	1 (2.8%)	0
Autoimmune thyroiditis	1 (2.8%)	0
Immune-mediated hepatitis	1 (2.8%)	1 (2.8%)
Immune-mediated hepatic disorder	1 (2.8%)	1 (2.8%)
Pyrexia	1 (2.8%)	1 (2.8%)
Asthenia	1 (2.8%)	0
Generalized edema	1 (2.8%)	0
Blood thyroid stimulating hormone increased	1 (2.8%)	0
Hypersensitivity	1 (2.8%)	0
Immune-mediated dermatitis	1 (2.8%)	0
Pruritus	1 (2.8%)	0
Cheilitis	1 (2.8%)	0

Data are shown in n (%). No grade 4 or 5 immune-related adverse events occurred.
